# The effectiveness of the first dose COVID-19 booster vs. full vaccination to prevent SARS-CoV-2 infection and severe COVID-19 clinical event: a meta-analysis and systematic review of longitudinal studies

**DOI:** 10.3389/fpubh.2023.1165611

**Published:** 2023-06-01

**Authors:** Junjie Xu, Xinquan Lan, Liangyuan Zhang, Xiangjun Zhang, Jiaqi Zhang, Moxin Song, Jiaye Liu

**Affiliations:** ^1^Clinical Research Academy, Peking University Shenzhen Hospital, Peking University, Shenzhen, China; ^2^Department of Epidemiology, School of Public Health, China Medical University, Shenyang, China; ^3^Department of Clinical Pharmacy and Translational Science, College of Pharmacy, University of Tennessee Health Science Center, Memphis, TN, United States; ^4^School of Public Health, Shenzhen University Medical School, Shenzhen, China

**Keywords:** COVID-19, SARS-CoV-2, vaccine, booster, effectiveness, meta-analysis

## Abstract

**Background:**

The effectiveness of full Coronavirus Disease 2019 (COVID-19) vaccination against COVID-19 wanes over time. This study aimed to synthesize the clinical effectiveness of the first dose of COVID-19 booster by comparing it to the full vaccination.

**Methods:**

Studies in PubMed, Web of Science, Embase, and clinical trials databases were searched from 1 January 2021 to 10 September 2022. Studies were eligible if they comprised general adult participants who were not ever or currently infected with SARS-CoV-2, did not have impaired immunity or immunosuppression, and did not have severe diseases. The seroconversion rate of antibodies to S and S subunits and antibody titers of SARS-CoV-2, frequency, phenotype of specific T and B cells, and clinical events involving confirmed infection, admission to the intensive care unit (ICU), and death were compared between the first booster dose of COVID-19 vaccination group and full vaccination group. The DerSimonian and Laird random effects models were used to estimate the pooled risk ratios (RRs) and corresponding 95% confidence intervals (CIs) for the outcomes of clinical interest. While a qualitative description was mainly used to compare the immunogenicity between the first booster dose of COVID-19 vaccination group and full vaccination group. Sensitivity analysis was used to deal with heterogenicity.

**Results:**

Of the 10,173 records identified, 10 studies were included for analysis. The first dose COVID-19 booster vaccine could induce higher seroconversion rates of antibodies against various SAS-CoV-2 fragments, higher neutralization antibody titers against various SARS-CoV-2 variants, and robust cellular immune response compared to the full vaccination. The risk of SARS-CoV-2 infection, the risk of admission to the ICU, and the risk of death were all higher in the non-booster group than those in the booster group, with RRs of 9.45 (95% CI 3.22–27.79; total evaluated population 12,422,454 vs. 8,441,368; *I*^2^ = 100%), 14.75 (95% CI 4.07–53.46; total evaluated population 12,048,224 vs. 7,291,644; *I*^2^ = 91%), and 13.63 (95% CI 4.72–39.36; total evaluated population 12,385,960 vs. 8,297,037; *I*^2^ = 85%), respectively.

**Conclusion:**

A homogenous or heterogeneous booster COVID-19 vaccination could elicit strong humoral and cellular immune responses to SARS-CoV-2. Furthermore, it could significantly reduce the risk of SARS-CoV-2 infection and severe COVID-19 clinical events on top of two doses. Future studies are needed to investigate the long-term clinical effectiveness of the first booster dose of the COVID-19 vaccine and compare the effectiveness between homogenous and heterogeneous booster COVID-19 vaccination.

**Systematic review registration:**

https://inplasy.com/inplasy-2022-11-0114/, identifier: INPLASY2022110114.

## 1. Introduction

As of 23 December 2022, Coronavirus Disease 2019 (COVID-19) caused 651.9 million cases and 6.7 million deaths globally (1). As a major prevention strategy, COVID-19 vaccination plays a vital role in reducing rates of mortality and severe events during this pandemic. The booster COVID-19 vaccine promoted humoral and cellular immunity through the recall of memory B cells, the *de novo* activation of B cells, and B cell maturation through the activation and development of follicular helper T (Tfh) cells. These were the physiopathology basis for minimizing the risk of COVID-19 infection and progression to severe diseases (2). The first dose of COVID-19 booster vaccination has been promoted worldwide to strengthen the effect of the full COVID-19 vaccination. As of 28 December 2022, the global booster administration rate was 33.5% (1). Previous research has investigated the effects of the COVID-19 vaccine booster on disease prevention. Various studies, including randomized controlled clinical trials (RCTs), used cell immunity and humoral immunity parameters to indicate the potential preventive effects of COVID-19, but no direct prevention effects have been discovered to date (3, 4). More clinical indicators have been recommended in the evaluation of the effects of the COVID-19 vaccine booster, such as the SARS-CoV-2 incidence rate, the effect on the prevention of severe COVID-19 disease, hospitalization, intensive care unit (ICU) admission, and mortality. The inclusion of these factors could provide a more objective and complete picture of the effects of the COVID-19 vaccine booster. Except for a few developed countries, most countries in the world are still vaccinated for wild SARS-CoV-2 strains. With the frequent emergence of mutant strains, it is urgent and crucial to determine whether the COVID-19 vaccines for wild strains can also prevent mutant SARS-CoV-2 strains in the real world. The evidence could provide vital guidance on the administration of COVID-19 vaccine boosters and inform associated COVID-19 prevention strategies locally and nationally. At present, there are two major types of COVID-19 vaccine booster approaches, namely heterologous booster vaccination and homologous booster vaccination (based on two shots of COVID-19 vaccine, injecting the first shot of booster vaccination with the same technical route vaccine is defined as homologous booster vaccination, or then injecting a first shot of booster vaccination with different technical routes vaccine is defined heterogeneous booster vaccination). Heterologous COVID-19 vaccine booster strategy has been promoted in many developing countries, including China (5, 6). However, the literature lacks systematic reports on the clinical effects of the heterologous COVID-19 vaccination on the prevention of SARS-CoV-2 infections (3, 6).

A recent meta-analysis study compared the preventive efficacy of the first dose of the COVID-19 booster with less than three doses of COVID-19 vaccination (7). Nevertheless, the preventive efficacy between the first dose of the COVID-19 booster and only two doses of the COVID-19 vaccination remained unclear. In addition, the studies that this meta-analysis synthesized included various research designs, including, RCTs, cohort studies, cross-sectional studies, and case–control studies (7). The findings must be more convincing when only studies with study designs of interest were chosen for the review. With full doses becoming the majority of the vaccinated population, it was necessary to investigate the effects of the COVID-19 vaccine booster by comparing it to the full doses of the COVID-19 vaccination. This pooled meta-analysis aimed to synthesize the literature of cohort studies and RCTs on COVID-19 booster efficacy compared to full doses (two doses) of COVID-19 vaccination by comparing the differences in the incidence of COVID-19, hospitalization rate, ICU rate, and mortality rate. With approximately two-thirds of the world's population not received the first dose of the COVID-19 vaccine booster, the findings of the study have implications on eliminating the hesitation of COVID-19 booster administration, increasing the awareness of COVID-19 booster vaccination, and providing robust evidence on COVID-19 booster promotion.

## 2. Methods

This systematic review was reported according to the Preferred Reporting Items for Systematic Reviews and Meta-Analyses (PRISMA) guidelines (8). We register this review to the INPLASY register. The registration number is INPLASY2022110114.

### 2.1. Literature search strategy

Between 1 January 2021 and 10 September 2022, we searched relevant studies that were published in PubMed, Embase, Web of Science, and clinical trials databases using a combination of comprehensive keywords, including “COVID-19,” “SARS-CoV-2,” “vaccination,” “vaccine,” “third,” “boost,” and “four” with Boolean operators and MeSH terms (Supplementary Table 1 for search strategy). We also searched relevant systematic reviews to add additional eligible studies. The searching, reviewing, and selecting literature were independently and blindly performed by two authors (Lan X and Zhang L). Discrepancies were resolved through consultation with a third author (Song M).

### 2.2. Study selection

Published articles were eligible for this study if they meet the following inclusion criteria: (1) observational studies (prospective or retrospective cohort) or RCTs with a minimum of 10 general adult participants in any study group; (2) at least involved one type of the booster COVID-19 vaccination after full vaccination (e.g., one dose of mRNA vaccine booster after two doses of mRNA vaccines); (3) a control group comprising participants who completed full COVID-19 vaccination but did not receive a booster; and (4) reported at least one of the outcomes of interest in both the booster group and full vaccination group with comparable time periods: serum antibodies against different SAS-CoV-2 fragments regardless of continuous or binary outcomes, cell-mediated immune, laboratory-confirmed infection, COVID-19-related hospitalization, COVID-19-related ICU admission, or death.

We did not include the following studies: (1) comprised participants who were ever or currently infected with SARS-CoV-2; (2) comprised participants who had impaired immunity or immunosuppression; (3) comprised participants who had severe diseases, such as patients who needed hemodialysis; (4) the studies did not have baseline data; (5) review studies; and (6) non-English publications.

### 2.3. Data extraction

We extracted the data according to a standardized form in Microsoft Excel 2016 (Microsoft Office, CA, USA). This process was also conducted by two authors independently and checked by a third author. The following study characteristics were collected: first author, study setting, year of publication, study design, and sample size. Other information was also summarized, such as participant characteristics comprising age and sex and immunization-related data including vaccine type and brand, the interval between prime full vaccination and booster vaccination, dosing schedule, and the number of participants who received each type of vaccine. Outcome-related data comprised the interval between booster vaccination and the assessed outcomes, antibody measured and the methods, frequency, and phenotype of specific T and B cells, mean or median of cytokine levels, and the number of events involving infection, hospitalization, admission to the ICU, and death.

### 2.4. Risk of bias assessment

We used the Risk of Bias in Non-randomized Studies of Interventions (*ROBINS-I*) for the quality assessment of all cohort studies, which consisted of seven domains: risk of bias from confounding, selection of participants, classification of interventions, deviations from intended interventions, missing data, measurement of outcomes, and selection of the reported results (9). In addition to cohort studies, we also included RCTs in this review. The risk of bias in RCTs was assessed using version 2 of the Cochrane risk-of-bias tool for randomized trials (10).

### 2.5. Statistical analysis

We performed all meta-analytical evaluations on R 4.0.3 using the *meta-packages*. We used the DerSimonian and Laird random effects model to estimate the pooled risk ratios (*RRs*) and corresponding 95% confidence intervals (*CI*s) for the outcomes of interest. *RR* was estimated as the event rate in the control group divided by the same rate in the booster group. We also estimated the summary vaccine effectiveness (VE) against various clinical outcomes. VE was obtained from the effect size (RR) defined as (1–1/RR) × 100%. Statistical heterogeneity was assessed using the Cochrane *Q* test and *I*^2^ statistics. We considered heterogeneity to be significant when the *p*-value was <0.10 or the *I*^2^ statistic was ≥50%. Unless specified otherwise, we considered a two-sided *p*-value of <0.05 to be statistically significant.

The techniques used to measure SARS-CoV-2 specific antibodies and criteria for positivity varied in different studies. Thus, meta-analysis was inappropriate to compare the antibody titers and seroconversion of different studies. Instead, a qualitative description was mainly used to compare and pool the immunogenicity.

Sensitivity analysis was used to deal with the heterogenicity by removing the studies with the highest effect value. The funnel plots, Egger's test, and Begg rank correlation test were used to assess the potential publication bias (11).

## 3. Results

### 3.1. Summary of included studies

**Figure 1:**
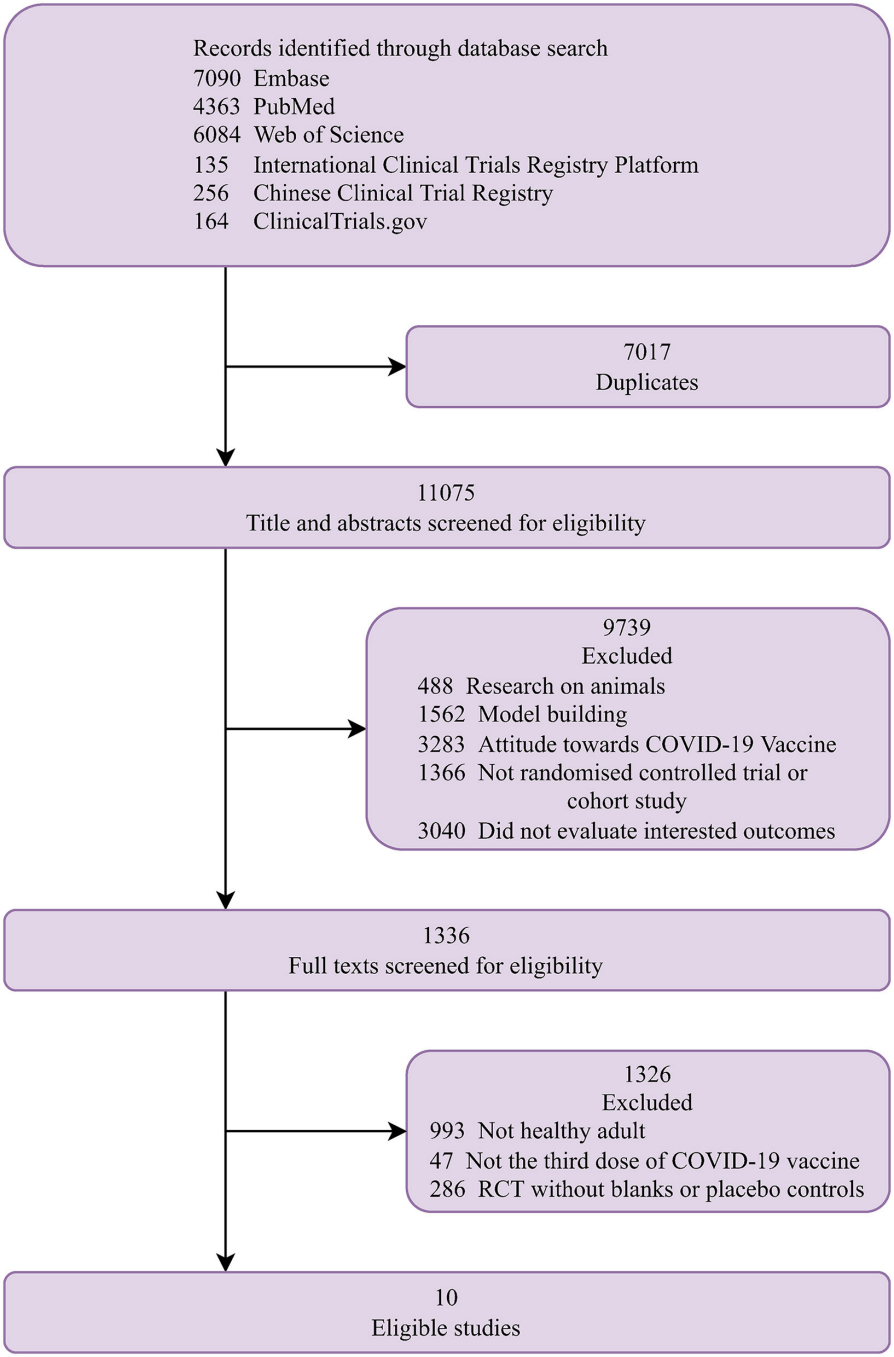
Study selection.

Figure 1 shows a flow chart of the study selection. Finally, 10 studies that met the eligibility criteria were included in the analysis (12–21). The characteristics of the included studies are summarized in Table 1. They were conducted one in each of the following countries: India (21), Singapore (20), Malaysia (16), Israel (13), UK (19), Qatar (12), Brazil (18), Abu Dhabi (17), Turkey (15), and China (14). The proportion of the female ranged from 7.1 to 78.5% and the median age ranged from 33 to 56 years. Five studies reported the proportion of comorbidities ranging from 7 to 75.4%. Of the 10 studies, one was a randomized trial (21) and nine were observational studies (12–20). Five studies included patients who received an mRNA (BNT162b2 or mRNA-1273) booster after the standard two-dose mRNA full vaccination (12, 13, 16, 19, 20); five studies included patients who received an inactivated booster vaccine after two-dose inactivated full vaccination (15–17, 20, 21); three studies included patients who received an mRNA booster vaccine after two-dose inactivated full vaccination (14–16); and one study included patients who received an mRNA-1273 booster vaccine after two-dose MVC-COV1901 vaccination (14). The qualitative analysis included the following studies: two studies reported the seroconversion rates of antibodies against different SAS-CoV-2 fragments (15, 21), three studies reported antibody titers (14, 17, 21), and two studies reported cell-mediated immune response after booster vaccination (14, 21). The meta-analysis included the following studies: six studies reported laboratory-confirmed SARS-CoV-2 infection (12, 13, 16, 18–20), three studies reported COVID-19-related ICU admission (16, 18, 20), and four studies reported mortality after that booster vaccination (12, 13, 16, 20).

**Table 1:** The characteristics of the included studies.

**References**	**Country/ region**	**Male (*n*, %)**	**Age (Years)**	**Comorbidities (*n*, %)**	**Study design**	**Boost Vaccine**	**Prime-boost interval (months)**	**Boost-outcome interval (days)**	**Comparison**
Vadrevuet al. (21)	India	3 Dose: 67/91 (73.6 %) 2 Dose with placebo: 73/93 (78.5%)	Median (IQR) 3 Dose: 35 (25–44) 2 Dose with placebo: 36 (26–44)	NR	RCT	BBV152 after 2 Dose BBV152	7.2 months	28	3 vs. 2 Dose BBV152 with placebo
Ng et al. (20)	Singapore	3 Dose: 1,058,786/2,201,604 (48.1%) 2 Dose: 103,748/239,977 (43.2%)	Median (IQR) 3 Dose: 53 (42–64) 2 Dose: 44 (36–60)	NR	Retrospective cohort study	mRNA after 2 Dose mRNA Inactivated vaccine after 2 Dose Inactivated vaccine	5 months (no later than 9 months)	15–60	3 Dose vs. 2 Dose mRNA 3 vs. 2 Dose inactivated vaccine
Low et al. (16)	Malaysia	3 Dose: 2,522,806/5,081,641 (49.6%) 2 Dose: 11,557,790/22,598,839 (51.1%)	Mean (SD) 39.9 (15.5)	4 451 180 (32.2)	Retrospective cohort study	BNT after 2 Dose BNT BNT after 2 Dose CoronaVac CoronaVac after 2 Dose CoronaVac	6 months after 2 dose BNT 3 months after 2 dose CoronaVac:	14	3 vs. 2 Dose BNT 3 vs. 2 Dose CoronaVac 2 Dose CoronaVac + BNT vs. 2 Dose BNT 2 Dose CoronaVac + BNT vs. 2 Dose CoronaVac
Arbel et al. (13)	Israel	3 Dose: 357,818/758,118 (47%) 2 Dose: 37118/85090 (44%)	Proportion of ≥65yr 3 Dose:470,808/758,118 (62) 2 Dose:35,208/85,090 (41) Proportion of 50–64 years 3Dose: 287,310/758,118 (38) 2 Dose: 49,882/85,090 (59)	NR	Prospetive cohort study	BNT after 2 Dose BNT	At least 5 months	7	3 vs. 2 Dose BNT
Menni et al. (19)	UK	3 Dose BNT: 70,699/20,4731 (34.5%) 2 Dose ChAd + BNT: 162,410/405,239 (40.1%) 2 Dose BNT + Mod: 70,699/20,4731 (34.5%) 2 Dose ChAd + Mod: 162,410/405,239 (40.1%) 2 Dose BNT: 70,699/204,731 (34.5%) 2 Dose ChAd: 162,410/405,239 (40.1%) 2 Dose Mod: 4,588/10,823 (42.4%)	Median (IQR) 3 Dose BNT: 52 (38–62) 2 Dose ChAd + BNT: 56 (46–63) 2 Dose BNT + Mod: 52 (38–62) 2 Dose ChAd + Mod: 56 (46–63) 2 Dose BNT: 52 (38–62) 2 Dose ChAd: 56 (46–63) 2 Dose mRNA-1273: 39 (33–46)	3 Dose BNT: 41,136/20,4731 (20.1%) 2 Dose ChAd + BNT: 66,471/40,5239 (16.4%) 2 Dose Mod: 755/10,823 (7%)	Prospetive cohort study	BNT after 2 Dose BNT BNT after 2 Dose ChAd Mod after 2 Dose BNT Mod after 2 Dose ChAd	NA	150–240	3 Dose mRNA vs. 2 Dose BNT 3 Dose mRNA vs. 2 Dose ChAd 2 Dose ChAd + mRNA vs. 2 Dose BNT 2 Dose ChAd + mRNA vs. 2 Dose ChAd
Abu-Raddad et al. (12)	Qatar	3 Dose BNT: 122,435/189,483 (64.6%) 3 Dose mRNA-1273: 45,443/66,191 (68.7%) 2 Dose BNT: 122,435/189,483 (64.6%) 2 Dose mRNA-1273: 45,443/66,191 (68.7%)	Median (IQR) 3 Dose BNT: 41 (34–50) 3 Dose mRNA-1273: 39 (33–46) 2 Dose BNT: 41 (34–50) 2 Dose mRNA-1273: 39 (33–46)	NR	Retrospective cohort study	BNT after 2 Dose BNT Mod after 2 Dose Mod	Median (IQR) BNT: 8.3 (7.7–9) Mod: 7.7 (7.4–8.3)	Median (IQR) BNT: 22 (12–28) Mod: 18 (8–32)	3 vs. 2 Dose BNT 3 vs. 2 Dose Mod
Marra et al. (18)	Brazil	2 Dose CoronaVac + BNT: 1,183/4,472 (26.5%) 2 Dose ChAd + BNT: 1,161/3,927 (29.6%) 2 Dose CoronaVac: 327/1,157 (28.3%) 2 Dose ChAd: 601/1,871 (32.1%)	Median (IQR) 2 Dose CoronaVac + BNT: 37 (31–43) 2 Dose ChAd + BNT: 37 (30–43) 2 Dose CoronaVac: 34 (28–40) 2 Dose ChAd: 33 (26–40)	2 Dose CoronaVac + BNT: 879/4,472 (25.7%) 2 Dose ChAd + BNT: 1,016/3,927 (29.4%) 2 Dose CoronaVac: 177/1,157 (21.2%) 2 Dose ChAd: 344/1,871 (24.7%)	Retrospective cohort study	BNT after 2 Dose CoronaVac or 2 Dose ChAd	NA	14	2 Dose Coronavac + BNT vs. 2 Dose Coronavac 2 Dose ChAd + BNT vs. 2 Dose ChAd
Mahmoud et al. (17)	Abu Dhabi	3 Dose BBIBP-CORV: 16/20 (66.1%) 2 Dose BBIBP-CORV: 20/35 (57.1%)	Mean (SD) 3 Dose BBIBP-CORV: 41.71 (9.86) 2 Dose BBIBP-CORV:41.84 (10.45)	NR	Retrospective cohort study	BBIBP-CORV after2 Dose BBIBP-CORV	NA	82	3 Dose vs. 2 Dose BBIBP-CORV
Demirhindi et al. (15)	Adana Turkey	3 Dose CoronaVac: 7.1% 2 Dose CoronaVac + BNT booster: 68.4% 2 Dose CoronaVac: 24%	Median (IQR) 3 Dose CoronaVac: 39.29 (8.18) 2 Dose CoronaVac + BNT booster: 40.67 (10.94) 2 Dose CoronaVac: 35.66 (8.13)	3 Dose CoronaVac: 6.6% 2 Dose CoronaVac + BNT booster: 75.4% 2 Dose CoronaVac: 18%	Prospetive cohort study	CoronaVac after 2 Dose CoronaVac BNT after 2 Dose CoronaVac	CoronaVac: 4.3–6 months BNT: 4.5–6.2 months	14	3 Dose vs. 2 Dose CoronaVac 2 Dose CoronaVac + BNT vs. 2 Dose CoronaVac
Chiu et al. (14)	Taiwan China	2 Dose MVC + Mod: 6/14 (42.9%) 2 Dose ChAd: 8/15 (53.3%) 2 Dose MVC: 6/14 (42.9%)	Median 2 Dose MVC + Mod: 44.5 2 Dose ChAd: 40 2 Dose MVC: 44.5	2 Dose MVC + Mod: 3/14 (21.4%) 2 Dose ChAd: 2/15 (13.3%) 2 Dose MVC: 3/14 (21.4%)	Prospetive cohort study	Mod after 2 Dose MVC	Median (IQR) 1.0 (0.97–1.07) months	14	2 Dose MVC + Mod vs. 2 Dose ChAd 2 Dose MVC + Mod vs. 2 Dose MVC

BNT, BNT162b2 (Pfizer-BioNTech); ChAd, ChAdx1-S (Oxford-AstraZeneca); Mod, mRNA-1273 (Moderna); MVC, MVC-COV1901; NA, not applicable; NR, not reported.

The quality assessment scores for included studies are shown in Supplementary Table 2. The randomized trial was considered of having some concerns of bias. Of the nine observational studies, seven studies (12, 13, 15, 16, 18–20), were considered to have a moderate risk of bias, one study (14) was at low risk of bias, and one study (17) was at serious risk of bias.

### 3.2. Seroconversion rates following booster shot

Two studies (15, 21) with three comparisons reported seroconversion rates after a booster dose. Vadrevu et al. (21) reported that one-dose booster of BBV152 induced higher seroconversion rates of antibody against spike protein (93.8 vs. 81.6%) and receptor binding domain (89.8 vs. 74.7%) and neutralization antibody by PRNT (98.7 vs. 79.8%) and MNT (100 vs. 92.9%) 1 month after the booster administration compared to full doses of the BBV152 vaccination. Moreover, after 6 months of the first booster dose administration, seroconversion of neutralization antibody against the D614G (96.8 vs. 59.5%), Delta (96.8 vs. 59.5%), and Omicron variant (93.5 vs. 56.8%) in the booster group was higher than those in the control group (21). Among individuals who received two doses of CoronaVac (Sinovac), both homologous vaccination (CoronaVac; 100 vs. 83.3%) and heterologous vaccination (BNT162b2; 100 vs. 83.3%) induced a higher seroconversion rate of antibody against receptor binding domain (15). In summary, these studies implied that booster vaccination could induce robust humoral response regardless of the booster vaccine type and anti-SARS-CoV-2 specific antibodies (Supplementary Table 3).

### 3.3. Antibody titers post-booster dose

Three studies (14, 17, 21) reported neutralization antibody titers against various SARS-CoV-2 variants. Vadrevu et al. (21) reported that the neutralization antibody titers of PRNT [746.2 (514.9–1,081) vs. 100.7 (43.6–232.6)] and MNT [641 (536.8–765.3) vs. 359.3 (267.4–482.7)] were higher in BBV152 booster group than those in the control group 1 month after the first booster dose administration. Moreover, after 6 months of the first booster administration, neutralization antibody titers against the D614G [178.9 (82.6–387.5) vs. 10.7 (2.6–44.5)], Delta [115.9 (55.8–240.8) vs. 7.3 (2.0–27.0)], and Omicron variants [25.7 (13.0–50.6) vs. 2.9 (0.99–8.3)] in the booster group maintained higher levels than those in the control group. Mahmoud et al. (17) reported that three doses of BBIBP-CorV induced higher neutralization antibody titers against SARS-CoV-2 Alpha (289.23 ± 186.30 vs. 138.46 ± 94.68), Beta (103.53 ± 62.94 vs. 34.12 ± 21.52), and Delta (156.89 ± 104.44 vs. 45.78 ± 29.96) variants compared with those received two doses of BBIBP-CorV. Similarly, Chiu et al. (14) reported that one-dose booster of mRNA-1273 after two doses of MVC-COV1901 induced 16.3-, 17.7-, and 32.2-fold higher neutralization antibody titers for the Alpha, Delta, and Omicron variants compared to those who received two doses of ChAdOx1-S and were 8.8-, 8.4-, and 26.0-fold higher than those received two doses of MVC-COV1901. In general, these studies suggested that the booster vaccination could induce higher neutralization antibody titers against various SARS-CoV-2 variants (Supplementary Table 4).

### 3.4. Cell-mediated immune post-booster dose

Two studies (14, 21) reported cell-mediated immunity. Vadrevu et al. (21) reported that the median Th1:Th2 index increased from 10.0 (IQR, 1.0–32.0) to 16.0 (IQR, 4.0–32.0) after the booster dose administration. The IFN-γ level was similar with a median of 48 (15.0–85.0) and 48 (29.0–95.0) in the booster and non-booster group, respectively, after 6 months of third-dose administration (21). Similarly, the median IgG secreting memory B cells (MBC) per 10^6^ PBMCs were increased in the booster group (50, IQR 12.0–60.0) compared to the non-booster group (21.3, IQR 14.2–43.5) (21). Chiu et al. (14) reported that the administration of an additional dose of mRNA vaccine after two doses of the subunit vaccine could significantly enhance the cellular immune response for both the wild type and the Delta variant (Supplementary Table 5).

### 3.5. Laboratory-confirmed infection, admission to the ICU, and death

**Figure 2:**
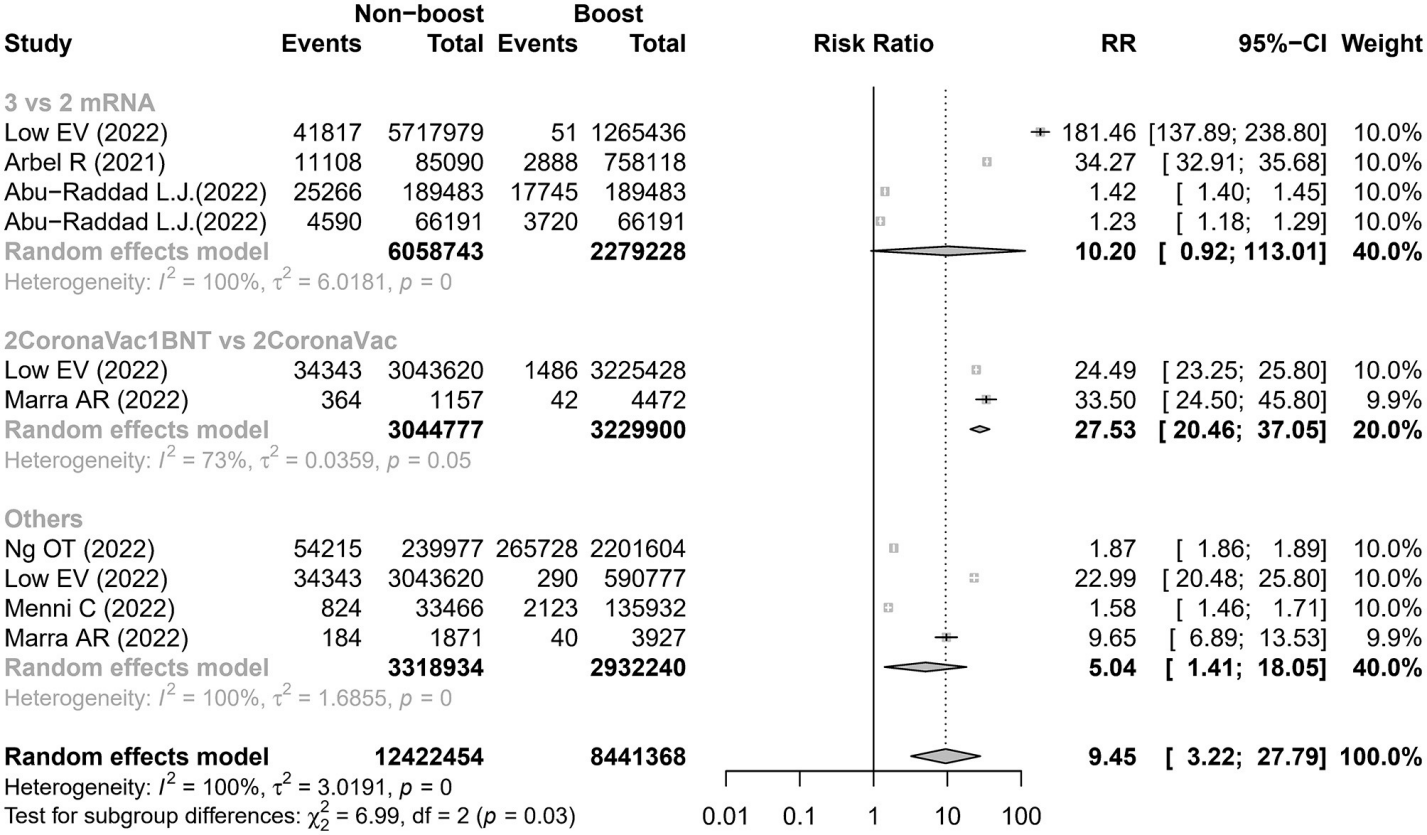
Estimates of the risk ratio of SARS-CoV-2 infection in the non-booster group vs. the booster group. BNT, BNT162b2(Pfizer-BioNTech); CI, confidence intervals; RR, risk ratio.

This meta-analysis found that the risk of SARS-CoV-2 infection was higher in the non-booster group than that in the booster group with a relative risk (RR) of 9.45 (95% CI 3.22–27.79) and with a summary of VE of 89.42% (95% CI 68.94%−96.40%), which was significantly heterogenous (I^2^ = 100%; Figure 2 and Table 2). We undertook further subgroup analyses to compare the results in the booster vaccination group and full doses vaccination group. A significant difference was found among the three groups (*p* < 0.05). The pooled RR of comparison between the two-dose inactivated vaccines vs. two-dose inactivated vaccines plus one-dose BNT162b2 was 27.53 (95% CI 20.46–37.05) with a summary VE of 96.37% (95% CI 95.11%−97.30%), which was higher than that in the comparison of two-dose mRNA vaccines vs. three-dose mRNA vaccines [10.20 (95% CI 0.92–113.01)]. Four studies (16, 18–20) used various prime and booster combinations, such as two doses of mRNA plus an mRNA booster, two doses of inactivated vaccine plus an inactivated booster in Ng et al.'s study (20), two doses of mRNA plus an mRNA booster and two doses of inactivated vaccine plus an inactivated booster or BNT162b2 in Low et al.'s study (16), two doses of mRNA or ChAdOx1-S plus an mRNA booster in Menni C's study (19), and two doses of ChAdOx1-S plus a BNT162b2 booster in Marra et al.'s study (18). However, three of these studies (18–20) did not report the number of SARS-CoV-2 infections associated with the specific booster vaccination. The pooled estimation also showed that individuals who received two doses of vaccination had a higher risk of SARS-CoV-2 infections than individuals who also received a booster vaccination (RR 5.04, 95% CI 1.41–18.05) with a summary VE of 80.16% (95% CI 29.08%−94.46%). After removing the studies with the highest effect value, sensitivity analysis gave similar results (Supplementary Figure 1).

**Table 2:** The prevention effect comparison of a third COVID-19 boost vaccines vs. the full COVID-19 vaccine.

**References**	**Country/ region**	**Diagnosis of COVID-19 infection**	**Boost vaccine comparison**	**SARS-CoV-2 infection (95% CI)**	**Admission to ICU (95% CI)**	**Death (95% CI)**
Vadrevuet al. (21)	India	NA	NA	NA	NA	NA
Ng et al. (20)	Singapore	Official COVID-19 database	Others^*^	RR: 1.87 (1.86, 1.89) VE: 0.47 (0.46, 0.47)	RR: 5.47 (3.42, 8.73) VE: 0.82 (0.71, 0.89)	RR: 13.99 (10.55, 18.55) VE: 0.93 (0.91, 0.95)
Low et al. (16)	Malaysia	Reverse transcription-polymerase chain reaction (RT-PCR) or rapid antigen test result	3 vs. 2 mRNA	RR: 181.46 (137.89, 238.80) VE: 0.99 (0.99, 1.00)	RR: 121.06 (7.55, 1,940.28) VE: 0.99 (0.87, 1.00)	RR: 80.78 (5.03, 1,296.34) VE: 0.99 (0.80, 1.00)
Low et al. (16)	Malaysia	Reverse transcription-polymerase chain reaction (RT-PCR) or rapid antigen test result	2 CoronaVac1BNT vs. 2 CoronaVac	RR: 24.49 (23.25, 25.80) VE: 0.959 (0.956, 0.961)	RR: 62.87 (35.59, 111.40) VE: 0.98 (0.97, 0.99)	RR: 82.92 (30.93, 222.33) VE: 0.99 (0.97, 1.00)
Low et al. (16)	Malaysia	Reverse transcription-polymerase chain reaction (RT-PCR) or rapid antigen test result	Others	RR: 22.99 (20.48, 25.80) VE: 0.96 (0.95, 0.96)	RR: 23.07 (10.33, 51.51) VE: 0.96 (0.90, 0.98)	RR: 20.25 (6.50, 63.13) VE: 0.95 (0.85, 0.98)
Arbel et al. (13)	Israel	Reverse-transcriptase–quantitative polymerase-chain-reaction (RT-qPCR)	3 vs. 2 mRNA	RR: 34.27 (32.91, 35.68) VE: 0.970 (0.969, 0.972)	NA	RR: 18.78 (13.98, 25.22) VE: 0.95 (0.93, 0.96)
Menni et al. (19)	UK	Self-reported lateral flow or PCR test	Others	RR: 1.58 (1.46, 1.71) VE: 0.37 (0.32, 0.42)	NA	NA
Abu-Raddad et al. (12)	Qatar	Polymerase chain-reaction (PCR) tests	3 vs. 2 BNT	RR: 1.42 (1.40, 1.45) VE: 0.30 (0.29, 0.31)	NA	RR: 2.88 (1.29, 6.43) VE: 0.19 (0.15, 0.22)
Abu-Raddad et al. (12)	Qatar	Polymerase chain-reaction (PCR) tests	3 vs. 2 mRNA-1273	RR: 1.23 (1.18, 1.29) VE: 0.65 (0.22, 0.84)	NA	RR: 1.00 (0.14, 7.10) VE: 0.00 (−6.14, 0.86)
Marra et al. (18)	Brazil	Reverse transcription–polymerase chain-reaction (RT-PCR) testing	2 CoronaVac1BNT vs. 2 CoronaVac	RR: 33.50 (24.50, 45.80) VE: 0.97 (0.96, 0.98)	RR: 15.46 (1.73, 138.19) VE: 0.94 (0.42, 0.99)	NA
Marra et al. (18)	Brazil	Reverse transcription–polymerase chain-reaction (RT-PCR) testing	Others	RR: 9.65 (6.89, 13.53) VE: 0.90 (0.85, 0.93)	RR: 1.05 (0.19, 5.72) VE: 0.05 (-4.26, 0.83)	NA
Mahmoud et al. (17)	Abu Dhabi	NA	NA	NA	NA	NA
Demirhindi et al. (15)	Adana Turkey	NA	NA	NA	NA	NA
Chiu et al. (14)	Taiwan China	NA	NA	NA	NA	NA

^*^The used booster vaccine includes the inactivated vaccine, or other types of COVID-19 vaccine (the homologous or heterologous vaccines).

NA, not applicable; Mod, mRNA-1273 (Moderna); BNT, BNT162b2 (Pfizer-BioNTech); VE, 1–1/RR.

**Figure 3:**
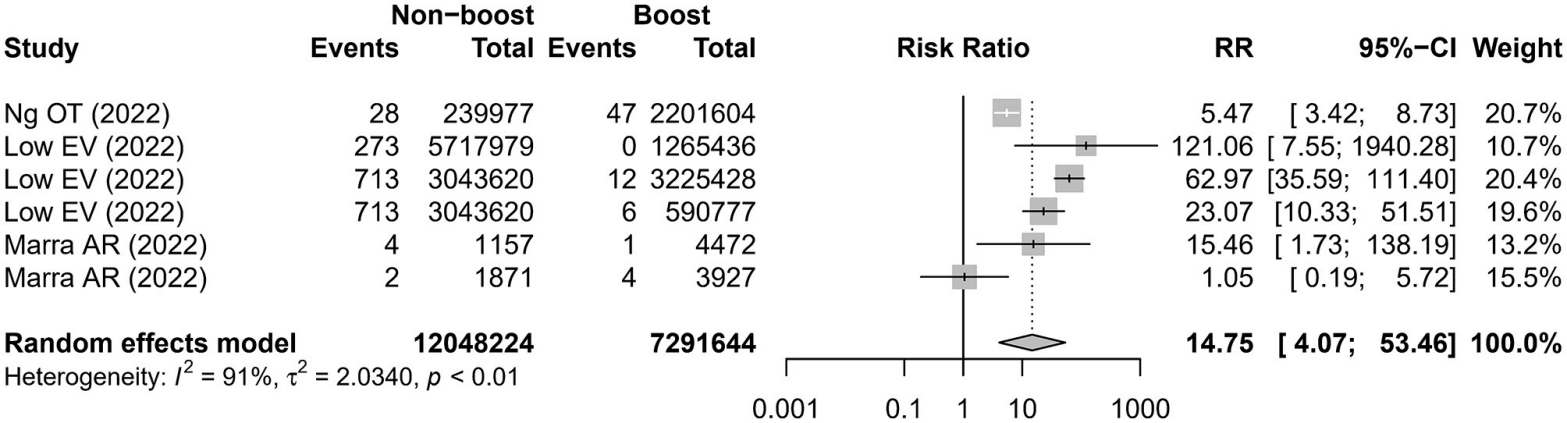
Estimates of the risk ratio of admission to the ICU in the non-booster group vs. in the booster group. CI, confidence intervals; RR, risk ratio.

**Figure 4:**
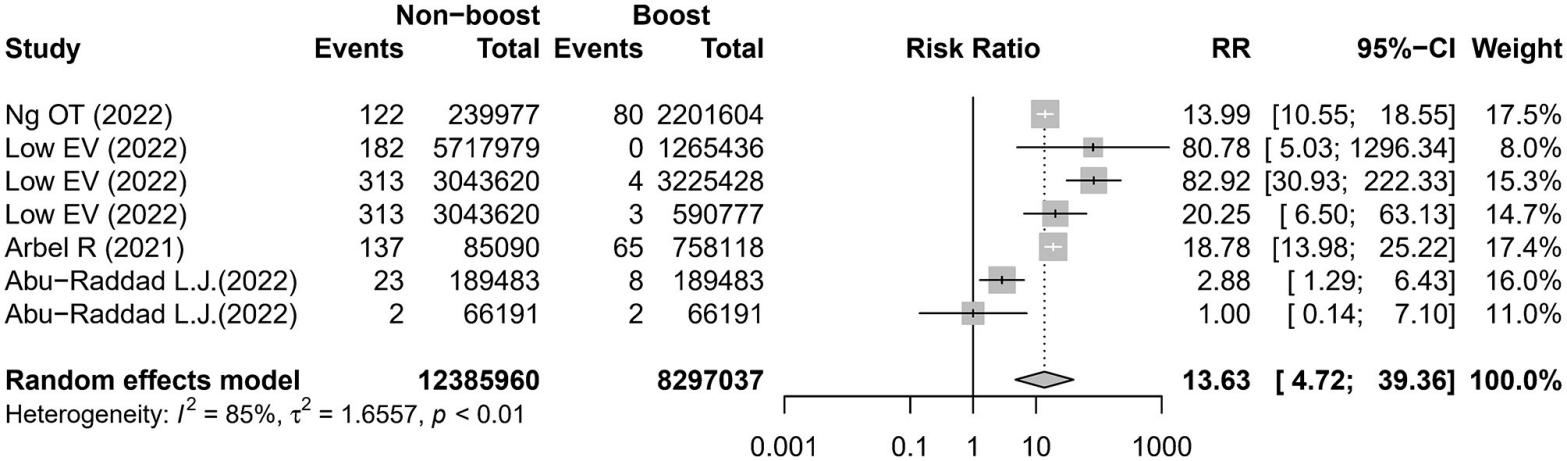
Estimates of the risk ratio of death in the non-booster group vs. in the booster group. CI, confidence intervals; RR, risk ratio.

Three studies (16, 18, 20) reported six comparisons on the admission rate to ICU. The meta-analysis showed that the risk of admission to the ICU was higher in the non-booster group than that in the booster group with an RR value of 14.75 (95% CI 4.07–53.46), which was significantly heterogeneous (I^2^ = 91%; Figure 3 and Table 2). After removing the studies with the highest effect value, sensitivity analysis gave similar results (Supplementary Figure 2). Four studies (12, 13, 16, 20) reported seven comparisons on the rate of death. The meta-analysis found that the risk of death was higher in the non-booster group than that in the booster group with an RR value of 13.63 (95% CI 4.72–39.36), which was significantly heterogenous (I^2^ = 85%; Figure 4 and Table 2). After removing the studies with the highest effect value, sensitivity analysis gave similar results (Supplementary Figure 3). Due to the limited number of included studies, we did not take further subgroup analyses on admission to the ICU and death by prime and booster vaccination group. No significant statistical publication bias was detected by Egger's test (p = 0.075, 0.852, and 0.993, respectively), Begg rank correlation test (p = 0.380, 0.0.719, and 1.000, respectively), and funnel plot (Supplementary Figures 4–6).

### 3.6. Heterogeneity

The *p*-value for Cochrane's *Q*-test suggested high heterogeneity across studies in the assessment of all events.

## 4. Discussion

COVID-19 remains a public health concern worldwide, and hence clarifying and optimizing the vaccination effects is urgently needed to guide disease prevention and control. This systematic review and meta-analysis found that the provision of a booster COVID-19 vaccination induced a higher seroconversion rate and antibody levels compared to the primary vaccination alone. Furthermore, this study showed that a booster COVID-19 vaccination resulted in the improvement of some indicators, including SARS-CoV-2 infection, admission to the ICU, and death, which reflected a substantial clinical protective efficacy of a booster vaccination. This systematic review provided comprehensive and solid evidence supporting the promotion of one booster dose COVID-19 vaccine in the general adult population on top of the full vaccination.

SARS-CoV-2 infection begins when the RBD of the S protein of the virus binds to the angiotensin-converting enzyme 2 (ACE-2) receptor in human cells. Thus, positive serologic tests for the vaccine antigenic target (S and S subunits, including RBD) or antibody titers have been regarded as the most useful surrogate endpoint for COVID-19 vaccine effectiveness. However, it has been proven that the effectiveness of COVID-19 vaccines wanes around 4–6 months after the primary series of vaccination has been completed (22, 23). Moreover, newly emerging variants of SARS-CoV-2 resulted in break-through viral infections in vaccinated individuals and recovered patients (24, 25). A booster dose of COVID-19 vaccine could be a promising strategy by inducing a higher seroconversion rate of vaccine antigenic target and a higher level of antibody titers. Four studies included in this systematic review found that a booster dose of the COVID-19 vaccine could induce a higher seroconversion rate of antibodies or higher level of antibody titers among individuals compared to those who only received full COVID-19 vaccination (14, 15, 17, 21). Specifically, two of the four studies showed that a booster dose increased the neutralization efficiency against the Alpha, Delta, and Omicron variants (14, 21). Among the two, one study investigated an inactivated vaccine booster vaccination after two-dose inactivated full vaccination (21) and the other was involved in an mRNA-1273 booster vaccine after two-dose MVC-COV1901 vaccination (14). It suggested that a booster vaccine regardless of whether it was heterologous or homologous with the prime vaccination had a good humoral response against various variants including Omicron, which was the dominant strain globally to date.

T cells can recognize viral protein segments of deep hid and are less susceptible to immune evasion via mutation, even for variants that were considered able to escape neutralizing antibodies (26, 27). Chiu et al. (14) found that the administration of an additional dose of mRNA vaccine after two doses of the subunit vaccine can significantly enhance the cellular immune response for both the wild type and the Delta variant. In addition, Vadrevu et al. (21) showed that IgG-secreting memory B cells were higher in the booster arm compared to the non-booster arm. These results implied that the provision of a booster COVID-19 vaccination could elicit both strong humoral and cellular immune responses to SARS-CoV-2.

In this study, the seroconversion rate or antibody titers were used as indicators to reflect the immune responses to a vaccine, and they worked as proxies on the effects of the vaccine regarding infection rates and severity of COVID-19. At the same time, the infection rate, admission rate to ICU, and death were direct indicators reflecting the effectiveness of COVID-19 vaccination. The protection effect against SARS-CoV-2 infection and severe disease had been widely confirmed, but this effect decreased in months after the prime vaccination. Furthermore, the frequent emergence of various variants of SARS-CoV-2 could also result in reduced protection effects in vaccinated individuals. Our meta-analysis showed that a third dose of the COVID-19 vaccine could significantly reduce the risk of SARS-CoV-2 infection, admission to the ICU, and death, regardless of whether it was heterologous or homologous with the prime vaccination. In addition, three of 10 comparisons in SARS-CoV-2 infection (12, 20), one of five comparisons in admission to the ICU (20), and three of seven comparisons (12, 20) were assessed during the Omicron wave. Thus, these results provided the most updated evidence on the clinical effects of the COVID-19 booster vaccination. The global average proportion of COVID-19 vaccine booster administration rate was 33.5% as of 28 December 2022 (1). With a huge gap remaining between the actual booster vaccination coverage and the ideal coverage, the findings of this study could reduce the booster vaccination hesitancy and add solid evidence to the current World Health Organization (WHO) recommendations on adult booster vaccination (28).

This study has limitations. First, we only included studies with either longitudinal or RCT study design in this review, the effects of some potential factors might not be fully ruled out due to the inclusion of observational studies, such as age and comorbidities. The uneven status of baseline information between the exposed group and the non-exposed group may affect the analysis results between the exposure factors and the outcome time. Therefore, relevant limitations need to be considered when interpreting the research results. Second, only 10 studies were eligible and were included in the analysis. Some subgroup analyses comparing the effectiveness of various booster vaccination strategies were unable to be performed (e.g., heterologous and homologous with the booster vaccination and the time interval between the prime and booster vaccinations). Third, most of the included studies assessed the clinical outcomes during the Delta wave and only two studies during the Omicron wave. The findings for the Omicron variant could be strengthened when more studies are available reporting the effectiveness of the booster vaccination in the Omicron wave in the future. Four, the heterogeneity was high in the pooled estimation of clinical effectiveness, which may be due to the small number of included studies with different study designs, including the different types of used SARS-CoV-2 vaccines and different vaccination duration. Nonetheless, this meta-analysis synthesized the study results on COVID-19 booster effectiveness by comparing the first dose of COVID-19 booster and full vaccination. Most of the included studies had large samples, which ensured a large sample of participants as a whole for the meta-analysis. Five, most of the included studies assessed clinical outcomes during the Delta wave. While the Omicron wave is the current predominant variant, so further studies need to focus on the effectiveness of booster vaccinations during the Omicron wave and any subsequent variants that may emerge in the future. Finally, the number of included studies is small (10), which may limit the robustness of our study findings, so the generalizability of your results should be cautious.

## 5. Conclusion

This systematic review and meta-analysis indicated that both types of homogenous or heterogeneous booster COVID-19 vaccination could elicit both strong humoral and cellular immune responses to SARS-CoV-2. Furthermore, a third dose of the COVID-19 vaccine could significantly reduce the risk of SARS-CoV-2 infection, admission to the ICU, and death. These results were also applicable to the Omicron variant. Future studies were needed to investigate the long-term clinical effectiveness of the first booster dose of the COVID-19 vaccine and compare the effectiveness between homogenous and heterogeneous booster COVID-19 vaccination.

## Data availability statement

The raw data supporting the conclusions of this article will be made available by the authors, without undue reservation.

## Author contributions

JX and JL drafted the manuscript and analyzed the data. XL, LZ, and MS performed the searching, reviewing, and selecting of literature. JL, XL, LZ, JZ, MS, and XZ collected the epidemiological data. JL and XZ designed and revised the manuscript. All authors have read and approved the final manuscript.
