# Supplementary material for: The effectiveness of the first dose COVID-19 booster vs. full vaccination to prevent SARS-CoV-2 infection and severe COVID-19 clinical event: a meta-analysis and systematic review of longitudinal studies
Source: Front Public Health. 2023 Jun 1;11:1165611. doi: 10.3389/fpubh.2023.1165611 (PMC10267329; doi:10.3389/fpubh.2023.1165611)
Supplement: Supplementary file 1 [file Data_Sheet_1.docx]

Supplementary Table 1: Search strategy

| Database | Search terms | Results |
| --- | --- | --- |
| PubMed | ((covid[Title]) OR (sars-cov-2[Title])) and ((third[Title/Abstract]) or (three[Title/Abstract]) or (four*[Title/Abstract]) or (boost[Title/Abstract])) and ((vaccin*[Title/Abstract]) or (Vaccination[Mesh]) or (vaccines[Mesh])) AND (2021/1/1:2022/9/10[pdat]) AND ((humans[Filter]) NOT ("Case Reports"[Publication Type] OR "Editorial"[Publication Type] OR "Review"[Publication Type] OR "Meta-analysis"[Publication Type]) | 2863 |
| EMBASE | ("covid":ab,ti OR "sars-cov-2":ab,ti) AND ("third":ti,ab OR "three":ti,ab OR "four*":ti,ab OR "boost":ti,ab) AND ("vaccin*":ti,ab OR "vaccination"/exp OR "vaccine"/exp) AND (humans)/lim AND ("Article" OR "Article in press" OR "Conference paper" OR "Data paper" OR "Short survey" OR "Preprint")/lim AND (2021/1/1:2022/9/10)/lim | 3239 |
| Web of Science | "covid":ti AND ("third":ti,ab OR "three":ti,ab OR "four*":ti,ab OR "boost":ti,ab) AND ("vaccin*":ti,ab or "vaccination"/exp or "vaccine"/exp) NOT ("Case Reports":it OR "review":it OR "Abstract":it OR "editorial":it OR "letter":it OR "news":it OR "non-English":la) | 4071 |
| <https://clinicaltrials.gov/> | "covid-19": Condition or disease AND "vaccine": Intervention/treatment | 164 |
| <https://www.who.int/clinical-trials-registry-platform> | "covid-19": Condition AND "vaccine": Intervention | 135 |
| <https://www.chictr.org.cn/> | "covid-19": Condition AND "vaccine": Intervention | 256 |

Supplementary Table 2: Risk of bias assessment for a. randomized (RoB-2 tool) and b. observational (ROBINS-I tool) studies.

a.

| Author Year | Domain 1. Randomization process | Domain 2. Deviations from intended interventions | Domain 3. Mising outcome data | Domain 4. Measurement of the outcome | Domain 5. Selection of the reported result | Domain 6. Overall Bias |
| --- | --- | --- | --- | --- | --- | --- |
| Krishna Mohan Vadrevu 2022 | Low | Some concerns | Some concerns | Low | Low | Some concerns |

b.

| Author Year | Domain 1. Confounding factors | Domain 2. Selection of participants | Domain 3. Intervention classification | Domain 4. Deviation from intervention | Domain 5. Missing data | Domain 6. Measurement of outcome | Domain 7. Selection of reported result | ROBINS-I overall score |
| --- | --- | --- | --- | --- | --- | --- | --- | --- |
| Oon Tek Ng 2022 | Moderate | Low | Low | Low | Low | Low | Low | Moderate |
| Ee Vien Low 2022 | Moderate | Low | Low | Low | Low | Low | Low | Moderate |
| Ronen Arbel 2021 | Moderate | Low | Low | Low | Low | Low | Low | Moderate |
| Cristina Menni 2022 | Moderate | Low | Low | Low | Low | Low | Low | Moderate |
| Laith J Abu-Raddad 2022 | Moderate | Low | Low | Low | Low | Low | Low | Moderate |
| Alexandre R Marra 2022 | Moderate | Low | Low | Low | Moderate | Low | Low | Moderate |
| Sally Mahmoud 2022 | Serious | Low | Low | Moderate | Low | Moderate | Low | Serious |
| Hakan Demirhindi 2022 | Low | Low | Low | Low | Low | Moderate | Moderate | Moderate |
| Chun-Hsiang Chiu 2022 | Low | Low | Low | Low | Low | Low | Low | Low |

RoB-2: risk of bias-2 tool, ROBINS-I: Risk of Bias In Non-randomised Studies of Interventions tool

*ROBINS-I is a tool which assesses the risk of bias in non-randomised studies of interventions by looking into pre-intervention, intervention and post-intervention domains.

Studies with low risk of bias (no or one moderate concern in the included domains) are comparable to randomised controlled trials. Studies with moderate risk of bias (up to four moderate concerns in the included domains) can be characterised as credible but cannot considered comparable to a well performed randomised trial. Studies with serious risk of bias (at least one serious concern or multiple moderate concerns in the included domains) have important problems in the design. Studies with critical risk of bias (critical concerns or multiple serious concerns in the included domains) are too problematic to provide useful evidence on the intervention effect.

Supplementary Table 3: Seroconversion rates after a first booster dose of COVID-19 vaccine

| First Author | Immunoassay | Viruse | Threshold for positive response | N | Serocon rate of tiral | Serocon rate of control | Conclusions |
| --- | --- | --- | --- | --- | --- | --- | --- |
| Krishna MohanVadrevu | PNT and MNT for live virus neutralization;  ELISA for S1, RBD and N protein | D614G Alpha Beta Delta Delta plus Omicron | NR | 1 month:  Trial: 80  Control: 87 | Seroconversion rate (95% CI)  S1: 74/80; 93.8%(86.0-97.9) RBD: 71/80; 89.8%(79.7-94.7) PRNT: 79/80; 98.7(92.8-99.9) MNT: 80/80; 100(95.3-100) | Seroconversion rate (95% CI)  S1: 72/87; 81.6%(71.9-89.1) RBD: 65/87; 74.7%(64.3-83.4) PRNT: 70/87; 79.8(69.6-87.8) MNT: 81/87; 92.9(85.1-97.3) | Booster vaccination significantly increased seroconversion rate of neutralization and binding antibody compared with two dose vaccination |
| Krishna MohanVadrevu | PNT and MNT for live virus neutralization;  ELISA for S1, RBD and N protein | D614G Alpha Beta Delta Delta plus Omicron | NR | 6 months:  Trial: 31  Control: 37 | Seroconversion rate (95% CI)  D614G: 30/31; 96.8%(81.5-99.8) Delta: 30/31; 96.8%(81.5-99.8%) Omicron: 29/31; 93.5%(77.2-98.9) | Seroconversion rate (95% CI)   D614G: 22/37; 59.5%(42.2-74.8) Delta: 22/37; 59.5%(42.2-74.8) Omicron: 21/37; 56.8%(39.6-72.5) | Booster dose increased the neutralization efficiency against both homologous and heterologous variants compared with two dose vaccination |
| Hakan Demirhindi | CLIA for S-RBD-IgG, S, N protein | NR | Non-reactive: <1.00 AU/mL Be reactive: ≥1.00 AU/mL | 3 Dose CoronaVac: N=12  2 Dose CoronaVac: N=42 | RBD: 100(12/12) | RBD: 83.3(35/42) | Booster dose induced higher seroconversion to RBD than two dose vaccination |
| Hakan Demirhindi | CLIA for S-RBD-IgG, S, N protein | NR | Non-reactive: <1.00 AU/mL Be reactive: ≥1.00 AU/mL | 2 Dose CoronaVac+BNT booster: N=137  2 Dose CoronaVac: N=42 | RBD: 100(137/137) | RBD: 83.3(35/42) | Booster dose induced higher seroconversion to RBD than two dose vaccination |

CLIA, chemiluminescence immunoassay analyser; PRNT, Plaque-reduction neutralization test; MNT, Microneutralization Test; ELISA, Enzyme linked immunosorbent assay; NR, no reported.

Supplementary Table 4 Neutralization antibody titres after a first booster dose of COVID-19 vaccine

| First Author | Immunoassay | Viruse | N | Median (IQR) of trial | Median (IQR) of control | Conclusions |
| --- | --- | --- | --- | --- | --- | --- |
| Krishna MohanVadrevu | PNT and MNT for live virus neutralization | D614G Alpha Beta Delta Delta plus Omicron | Day 243:  Trial: 80  Control: 87 | Geometric mean titer (95% CI)  PRNT: 746.2(514.9-1081) MNT : 641.0(536.8-765.3) | Geometric mean titer (95% CI) PRNT: 100.7(43.6-232.6) MNT: 359.3(267.4-482.7) | A booster dose induced higher neutralization antibody titers compared with 2 dose vaccination |
| Krishna MohanVadrevu | PNT and MNT for live virus neutralization | D614G Alpha Beta Delta Delta plus Omicron | Day 395:  Trial: 31  Control: 37 | Geometric mean titer (95% CI)  D614G: 178.9(82.6-387.5) Delta: 115.9(55.8-240.8) Omicron: 25.7(13.0-50.6) | Geometric mean titer (95% CI)  D614G: 10.7(2.6-44.5) Delta: 7.3(2.0-27.0) Omicron: 2.9(0.99-8.3) | A booster dose induced higher neutralization antibody titers against both homologous and heterologous variants compared with 2 dose vaccination |
| Sally Mahmoud | PNT for live virus neutralization | Wild (Wuhan) strain Alpha Beta  Delta | Trial:20  Control:35 | The mean titers  Alpha:289.23 ±186.30 Beta:103.53 ± 62.94  Delta: 156.89 ± 104.44 | The mean titers  Alpha: 138.46 ± 94.68 Beta: 34.12 ±21.52  Delta: 45.78 ± 29.96 | The booster doses had better neutralizing effect against variants of concern compared with 2 dose vaccination |
| Chun-Hsiang Chiu | pVNT50 for neutralization assay with pseudotyped SARS-CoV-2 | Alpha  Delta  Omicron | 2 Dose MVC+Mod: 11  2 Dose AZ: 15  2 Dose MVC: 14 | The median(IQR) values(pVNT50)  Alpha: 4757.36 (4116.28-5958.05) U/mL Delta: 1142.49 (95980-1822.13) U/mL Omicron variants: 789.35 (587.39-1199.37) U/mL | The medians of pVNT50 with 2 Dose MVC+Mod for the Alpha, Delta, and Omicron were 16.3-, 17.7-, and 32.2-fold higher than those with 2 Dose AZ and 8.8-, 8.4-, and 26.0-fold higher than those with 2 dose MVC | Boosting with a first dose of Mod after priming with two doses MVC produced a stronger humoral response against the Alpha, Delta and Omicron |

Mod, mRNA-1273(Moderna); BNT, BNT162b2(Pfizer-BioNTech); ChAd, ChAdx1(Oxford-AstraZeneca); MVC, MVC-COV1901; AZ, AZD1222; PRNT, Plaque-reduction neutralization test; MNT,

Microneutralization Test; pVNT50, 50% pseudovirus neutralization titerm.

Supplementary Table 5 Cell-mediated immune outcome after a first booster dose of COVID-19 vaccine

| First Author | Cell-mediated immune outcome | N of trial | Median (IQR) of trial | N of control | Median (IQR) of control | Conclusions |
| --- | --- | --- | --- | --- | --- | --- |
| Krishna MohanVadrevu | Th1 and Th2 dependent immunoglobulin subclasses | Day 243  Trial: 80 | Day 215  Th1/Th2: 10.0 (1.0–32.0)  Day 243  Th1/Th2: 16.0(4.0-32.0) | Day 243  Control: 87 | NA | Th1 biased immune response is sustained after the booster dose. |
| Krishna MohanVadrevu | IFNγ T cell | Day 395  Trial: 31 | 48 (15.0–85.0) per 10^6 PBMCs | Day 395  Control: 37 | 48 (29.0–95.0) per 10^6 PBMCs | The SARS-CoV-2 T cell recall responses were found similar in booster and non-booster group |
| Krishna MohanVadrevu | Proportion of central and effector memory T cells among the SARS-CoV-2-specific CD4+ T cell population | Day 215: N=91 Day 243: N=80 | Day 215: CD4+ T cell T_CM_: 34.6%, T_EM_: 42.7%, T_EMRA_: 0.2% CD8+ T cell T_CM_: minimal, T_EM_: minimal, T_EMRA_: 26.9% Day 243: similar to Day 215 | Day 215: N=93 Day 243: N=87 | Day 215: CD4+ T cell T_CM_: 34.8%, T_EM_: 40.8%, T_EMRA_: 0.0%  CD8+ T cell T_CM_: minimal, T_EM_: minimal, T_EMRA_: 13.7%  Day 243: similar to Day 215 | A phenotypic profile of antigen specific CD4+and CD8+T cells associated with protective immunity to SARS-CoV-2 infection with a good antigen recall response |
| Krishna MohanVadrevu | SARS-CoV-2 specific antibody (IgG/IgA) memory B cells | Day 215: N=91 Day 243: N=80 | Day 215: IgG 20.5(13.5–34.8) per 10^6 PBMCs IgA 8.5(5.0-17.) per 10^6 PBMCs Day 243:  IgG 35.5(14.0-53.8) per 10^6 PBMCs IgA 6.5(0.0-25.5) per 10^6 PBMCs | Day 215: N=93 Day 243: N=87 | Day 215: IgG 13.0(0.0–20.0) per 10^6 PBMCs IgA 13.0(8.0-24.0) per 10^6 PBMCs Day 243:  IgG 28.0 (13.0-45.0) per 10^6 PBMCs IgA 10.0 (0.0-25.5) per 10^6 PBMCs | IgG secreting memory B cells remains elevated in booster arm over the non-booster arm |
| Chun-Hsiang Chiu | Gamma interferon (IFN-γ)-secreting T cell | 2 Dose MVC+Mod booster: N=11 | IFN-γ-secreting T cells The wild-type: 147.3(130.8-154.7) SFCs/10^6 PBMCs Delta variant: 120.6(104.3-140.2) SFCs/10^6 PBMCs | 2 Dose AZ: N=15  2 Dose MVC: N=14 | NA | Administration of an additional dose of mRNA vaccine after priming with two doses of the subunit vaccine can significantly enhance the cellular immune response for both the wild type and the Delta variant |
| Chun-Hsiang Chiu | The interleukin-2 (IL-2)-secreting T cell | 2 Dose MVC+Mod booster: N=11 | Smilar with IFN-γ-secreting T cell | 2 Dose AZ: N=15  2 Dose MVC: N=14 | NA | Administration of an additional dose of mRNA vaccine after priming with two doses of the subunit vaccine can significantly enhance the cellular immune response for both the wild type and the Delta variant |

Mod, mRNA-1273(Moderna); BNT, BNT162b2(Pfizer-BioNTech); ChAd, ChAdx1(Oxford-AstraZeneca); MVC, MVC-COV1901; PRNT, Plaque-reduction neutralization test; MNT, Microneutralization Test; pVNT50, 50% pseudovirus neutralization titer

Figure S1 Sensitivity analysis of estimates of risk ratio of SARS-CoV-2 infection in non-booster group versus in the booster group.


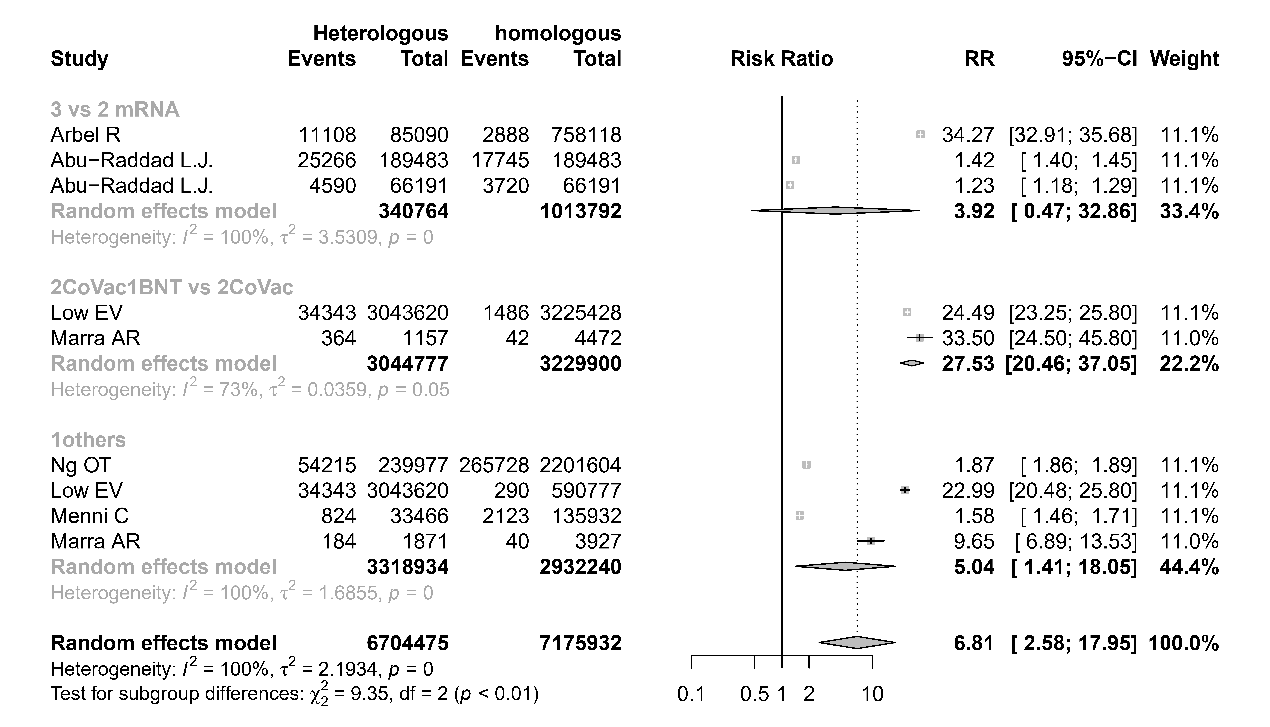


Figure S2 Sensitivity analysis of estimates of risk ratio of admission to the ICU in non-booster group versus in the booster group.


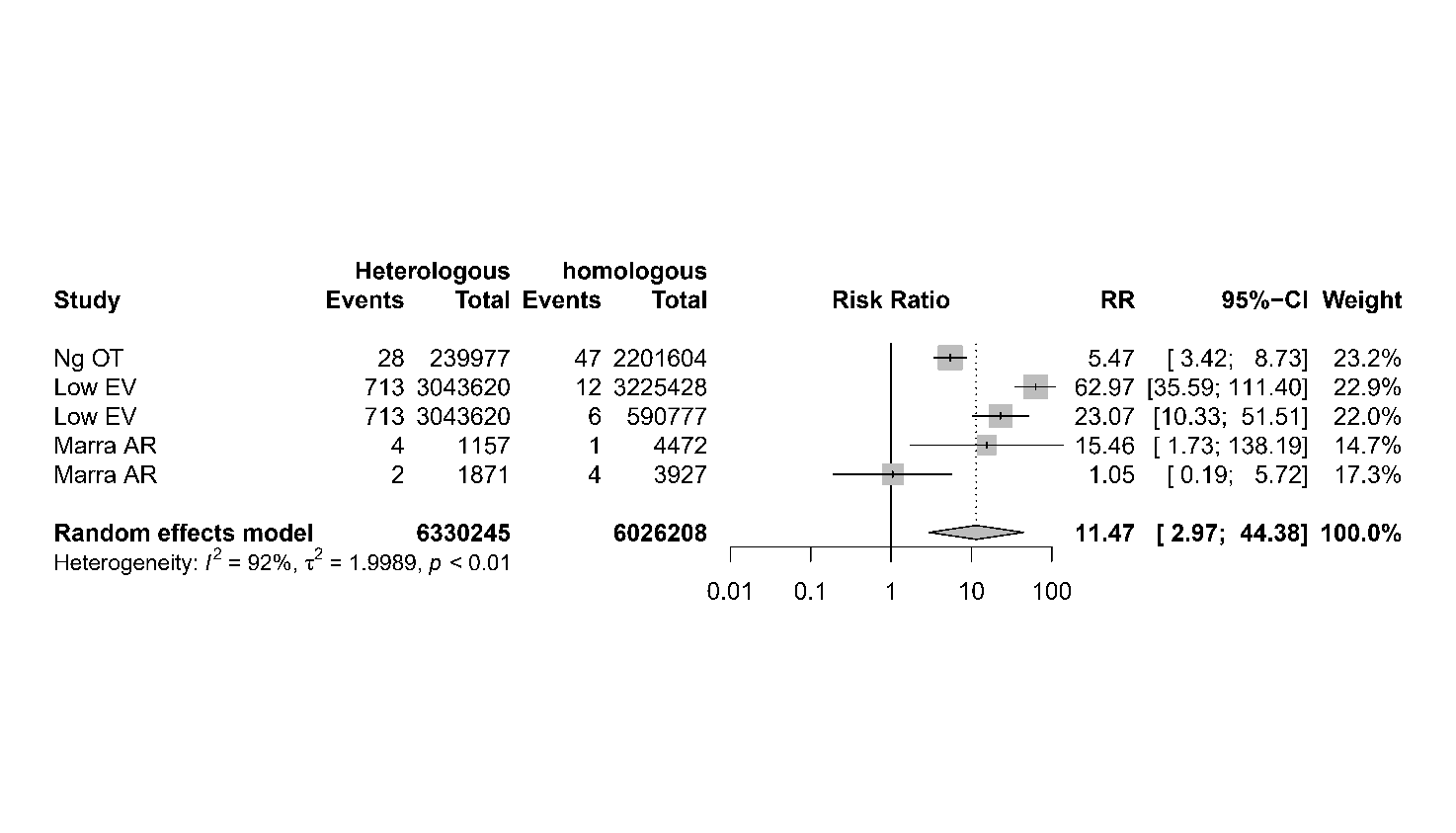


Figure S3 Sensitivity analysis of estimates of risk ratio of death in non-booster group versus in the booster group.


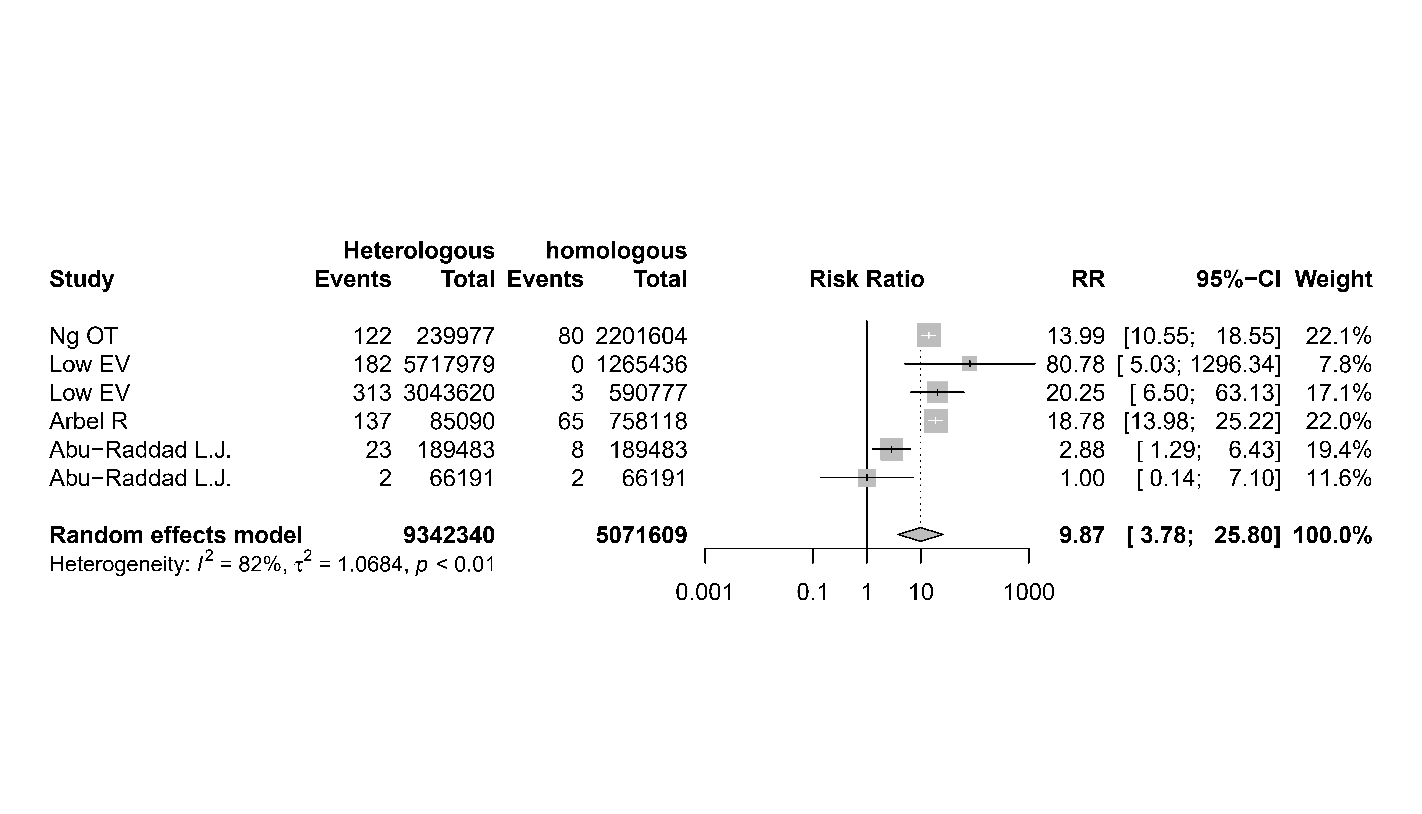


Figure S4 Funnel plot for assessment of publication bias for the outcome of the SARS-CoV-2 infection


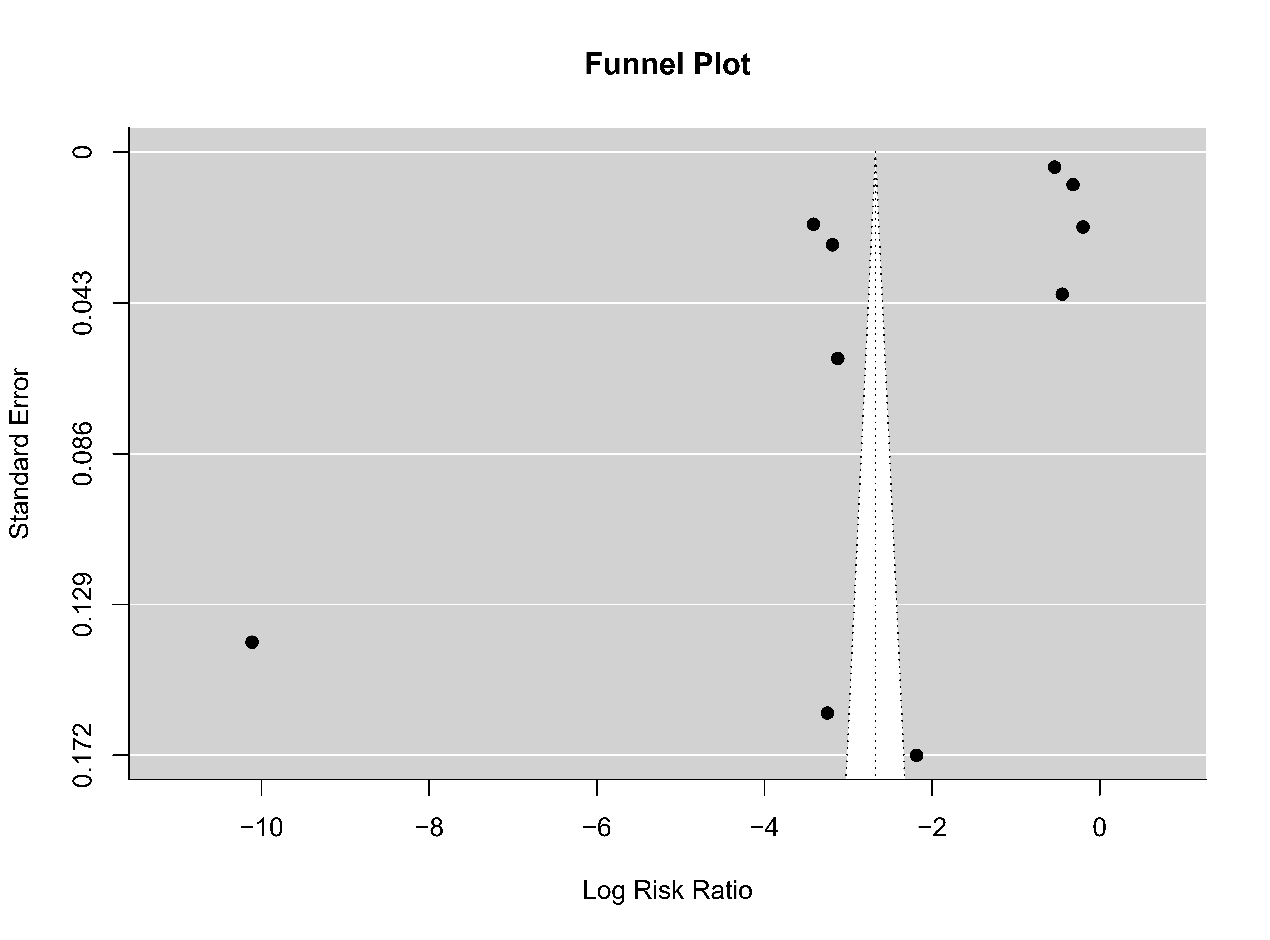


Egger’s test for funnel plot asymmetry: z = -1.7783, p = 0.0754

Begg rank correlation test for funnel plot asymmetry: Kendall's tau = -0.2444, p = 0.3807

Figure S5 Funnel plot for assessment of publication bias for the outcome of admission to ICU


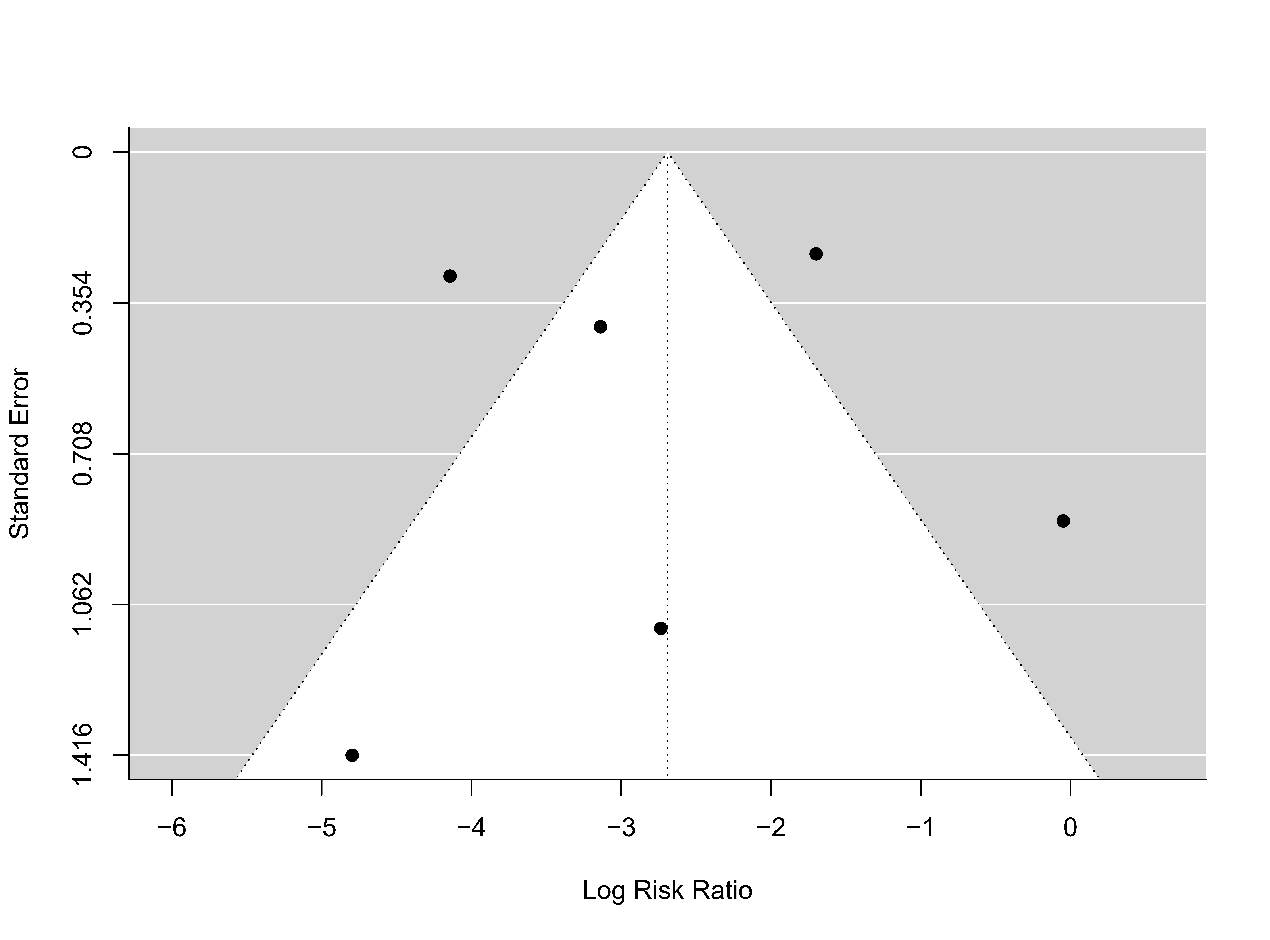


Egger’s test for funnel plot asymmetry: z = -0.1870, p = 0.8516

Begg rank correlation test for funnel plot asymmetry: Kendall's tau = -0.2000, p = 0.7194

Figure S6 Funnel plot for assessment of publication bias for the outcome of death


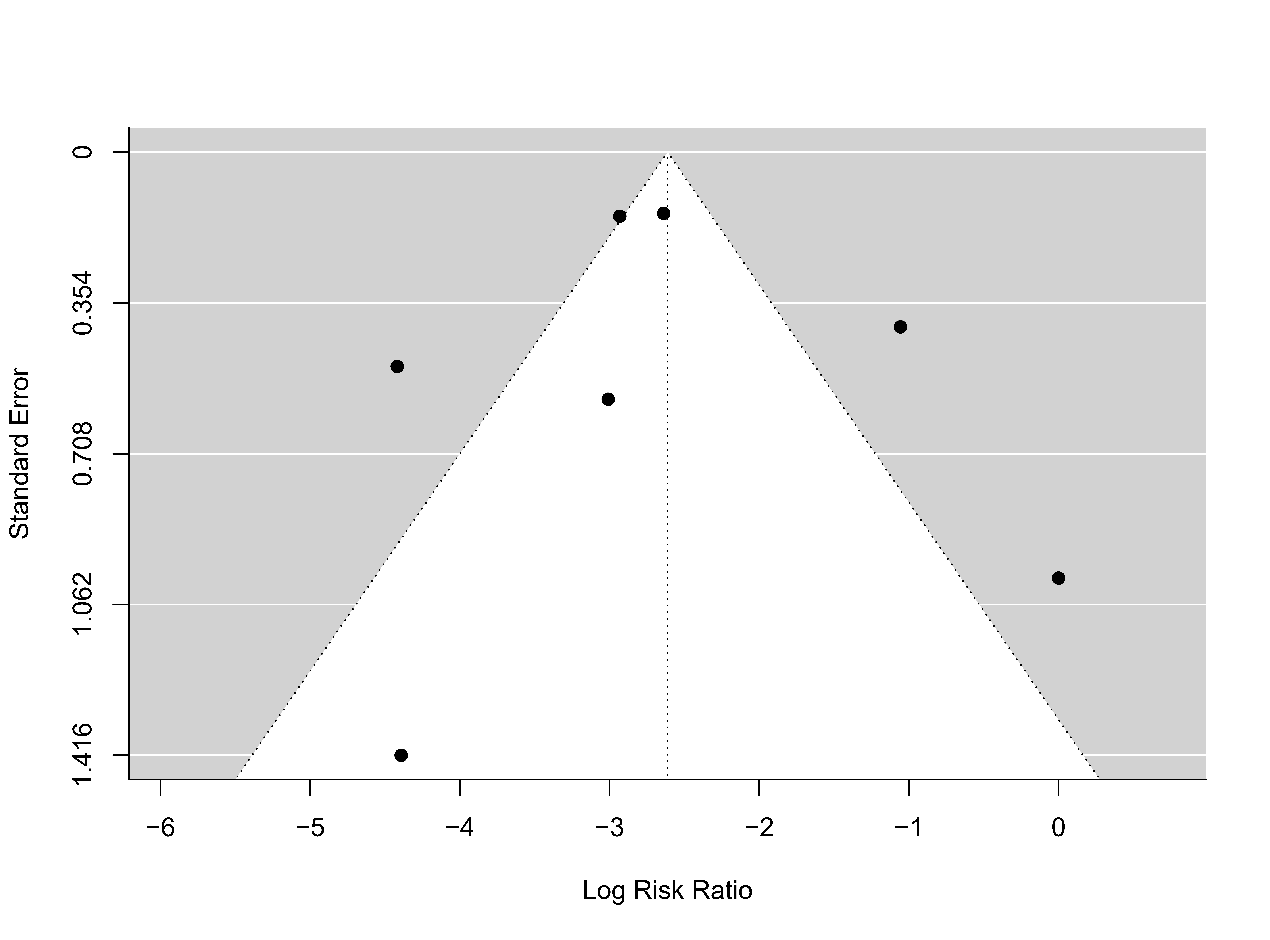


Egger’s test for funnel plot asymmetry: z = 0.0089, p = 0.9929

Begg rank correlation test for funnel plot asymmetry: Kendall's tau = -0.0476, p = 1.0000
